# The Growth and Scope of Voluntary Hospital in London

**Published:** 1897-07-24

**Authors:** 


### The Institutional Workshop.

### THE GROWTH AND SCOPE OF VOLUNTARY HOSPITALS IN LONDON.

(From a Correspondent.)

### I.—RISE OF THE VOLUNTARY SYSTEM.

The voluntary hospital system now in operation all over Great Britain owes nothing to enactments and little to theories. It was the outcome of urgent necessities acting on a pure spirit of human pity, and however complex the motives which have since been enlisted in its service, this vital principle has hitherto been strong enough to absorb and transmute all to good.

In the course of the two centuries during which the public has undertaken with voluntary offerings the care of the sick poor, hospital development in London has proceeded in a curiously unequal fashion. Although the great principle of continuity, which characterises all English institutions, has never failed; although each succeeding generation has loyally accepted the responsibility involved in the maintenance of existing hospitals, and added something at any rate towards the needs of the fast growing population; there have been periods of stagnation, alternating with the waves of enthusiasm, there have been blunders to repair, and anachronisms long uncorrected, as well as great achievements and epoch-making innovations. As might be expected in such an essentially popular growth, every phase of public feeling, every crisis in the national life, every new discovery, every time of general prosperity, or of famine, or of excitement, has had its influence and left its mark on London hospitals.

There has never been a time when these hospitals have been more efficient, more numerous, more widely supported, and more popular among those for whom they are intended than now. Consequently there has never been a time when their aims and work have been more severely criticised. It is idle to pass judgment upon hospital methods and hospital deficiencies apart from a study of the social condition of the community which supports and is benefitted by them. Rightly to appreciate the aims and understand the anomalies of the existing system, this study must stretch back beyond the limits of the century, and embrace the growth of the hospitals side by side with the spread of the vast aggregation of humanity to which they minister.

There is no need to recall the origin of the Royal hospitals. The history of St. Bartholomew's and St. Thomas's has been often told, and it is sufficient to remind the reader that they survived as a witness to the noble charity of early days, when many other rich endowments for the sick and poor were appropriated to private ends, and that, although more than once rescued from ruin by the rich gifts of the citizens, they were at no time dependent on popular support. Symptoms, however, of that revival of interest in hospital work which culminated in the great voluntary system, may be traced in the history of both the royal hospitals at the close of the 17th century. They had both happily escaped injury in the Great Fire, but by the destruction of much property supplying their revenues, their funds had suffered an alarming decrease. At the same time both hospitals were found to be in an almost ruinous condition. In this alarming crisis of their affairs, when London seemed likely to be left with absolutely no provision for the sick poor, a subscription was raised amongst the citizens, and by this means St. Bartholomew's was repaired in 1691, and the damaged house property restored to good condition; two years later St. Thomas's, by a like voluntary effort, was entirely rebuilt. The good initiated was carried on in the latter case by the addition of six new wards at the expense of private donors.

There was, perhaps, never a time when the condition of the poor in London more urgently called for the intervention and personal service of charitable persons than in the first decades of the eighteenth century. The population and wealth of the country had rapidly increased under the settled government inaugurated by William III. and continued under the Hanoverian dynasty. The rapid growth of London in particular is strikingly illustrated by the Bill passed in 1713 for the building of fifty new churches in the metropolis. The increase of wealth brought only into sharper contrast the desperate condition of the poor. London then, as now, a refuge for the unemployed, was crowded with discharged soldiers and seamen in every stage of want and disease; and from the scanty returns of the Poor Law expenditure it appears that those members of the population not in receipt of a living wage were out of all proportion to the body of regular working people. The gross and cruel abuses of the scanty privileges of the poor, and the absolute powerlessness of the law to restrain their oppressors, are painfully illustrated by the futile attempts at prison reform. The new and merciful regulations put forth after the Parliamentary inquiry of 1728 into the state of prisons, were almost universally disregarded, and, under the very eye of the law, incredible cruelty and injustice continued to be openly practised in these places throughout the country. The churchwardens and overseers of the poor had enough on their hands with the race of sturdy beggars which was perpetuated by the statutes enacted for their repression. Enormous sums were levied as poor-rate, but owing to a universal system of maladministration and peculation, little found its way to the right recipients. The workhouses established one after another in London in the course of the century apparently acted as a refuge for the vicious and dissolute in the last extremity of want, and as an habitual asylum for that backboneless class of paupers-in-grain, not yet extinct. The discipline and sanitary condition of these places, which were expected to partake of the double character of houses of correction and labour-homes, was unspeakably bad, and it is abundantly clear that they could afford no means of relief to the sick.

Under these circumstances, it is not surprising that charitable persons should have at length taken the matter into their own hands, and devised the best means in their power of lending aid, independent of the public funds, to the sick and suffering at their doors.

It is characteristic of the Londoner, whose benefactions have never known any distinction of race, that the first voluntary hospital regularly incorporated was that in the parish of St. Luke's, Middlesex, for poor French Protestants and their descendants residing in Great Britain. This institution, which owed its origin to a legacy bequeathed by Monsieur de Castigny, an attendant of William III., was founded in 1718, and its governors and directors formed by letters patent under the great seal into "a body corporate and politic for ever." The legacy proving far insufficient for the purpose, the project received liberal support from English sympathisers and the place was ultimately enlarged to contain as many as 200 sick, bedridden from age, or mentally afflicted. In the present day, when one of the most pressing perplexities of the social reformer is the tide of indigent foreign immigration flowing steadily Londonwards, it may not be amiss to recall this traditional London attitude of large-minded generosity towards the destitute and persecuted stranger.

The impulse to assist the foreigner in his exceptionally forlorn condition may have drawn attention to the needs of Englishmen. At any rate, the following year, 1719, saw the establishment of Westminster Hospital and the regular inauguration of the voluntary hospital system. The French Protestants aimed at the relief of one class only, and their institution was fully as much for the incurable and aged as for those suffering from acute disease. The Westminster Hospital proclaimed itself for the benefit of "the sick and needy from all parts." A small ward for incurables was added by separate endowment, but the main object of the institution was cure. Three of the peculiar features of the voluntary system, subscribers' letters, subscribers' control and faith in the future implied by dependence on yearly contributions, originated with the Westminster Hospital. A subscriber of £2 2s. could recommend two in and four out-patients in the year; this was the lowest qualification. A subscription of £3 3s. a year, or £30 down, constituted the subscriber a trustee, and all the business of the hospital was transacted by weekly or quarterly boards of trustees. These qualifications and privileges remain practically unaltered, and on this basis most of the hospitals subsequently founded have been framed.

The hospital did, in fact, owe its existence to actual knowledge on the part of its promoters of the condition of the poor in that particular neighbourhood. The recommendation signed by the subscriber was no idle form in days when every employer of labour had intimate knowledge of his men, when persons rarely shifted their residence and new-comers in a neighbourhood were eyed with suspicion. The provision for decently clothing in-patients, if necessary, shows that the benefits of the hospital were extended to the most destitute, but the rogue and vagabond belonged to a different set altogether. He would not be refused admittance if stabbed in some brawl near the hospital, for beds were from the first reserved for accidents, but a subscriber's letter would not be in the least likely to fall in his way, and pressure on space very early rendered indiscriminate admission impossible.

The same knowledge of the neighbourhood and its needs which rendered subscribers the fittest persons to recommend patients, rendered them also the fittest persons to control the affairs of the hospital. The mere fact of subscription to such an undertaking in those days implied an interest in the welfare of the poor and a degree of public spirit, qualifying for a share in its management. Many of the governors and trustees were men of tried business ability, and keen interest in the new project evoked from the beginning a healthy spirit of emulation among them for positions of influence in the administration. This principle was well indicated in one of the earliest reports issued of the hospital. "As all subscribers are trustees of this charity, so the more they will please to engage themselves in conducting the affairs of it, they will still be the greater benefactors to it, and will also be able to satisfy themselves that the money given is justly and frugally applied."

It would seem that no plan less liable to abuse could be devised than this, by which practical men, well acquainted with the needs of the people, undertook to organise, manage and defray the cost of an institution for supplying medical aid to their poorer brethren. Their faith that succeeding generations would carry on the work they inaugurated has seen an ample justification, but they had little conception of the mighty growth into which their germ would develop.

